# Response of Soil Respiration to Soil Temperature and Moisture in a 50-Year-Old Oriental Arborvitae Plantation in China

**DOI:** 10.1371/journal.pone.0028397

**Published:** 2011-12-06

**Authors:** Xinxiao Yu, Tianshan Zha, Zhuo Pang, Bin Wu, Xiaoping Wang, Guopeng Chen, Chunping Li, Jixin Cao, Guodong Jia, Xizhi Li, Hailong Wu

**Affiliations:** 1 Chinese Institute of Green Carbon, The School of Soil and Water Conservation, Beijing Forestry University, Beijing, China; 2 The Faculty of Forestry, Sichuan Agricultural University, Yaan, China; 3 Research Institute of Forest Ecology, Chinese Academy of Forestry, Beijing, China; Argonne National Laboratory, United States of America

## Abstract

China possesses large areas of plantation forests which take up great quantities of carbon. However, studies on soil respiration in these plantation forests are rather scarce and their soil carbon flux remains an uncertainty. In this study, we used an automatic chamber system to measure soil surface flux of a 50-year-old mature plantation of *Platycladus orientalis* at Jiufeng Mountain, Beijing, China. Mean daily soil respiration rates (*R_s_*) ranged from 0.09 to 4.87 µmol CO_2_ m^−2^s^−1^, with the highest values observed in August and the lowest in the winter months. A logistic model gave the best fit to the relationship between hourly *R_s_* and soil temperature (*T_s_*), explaining 82% of the variation in *R_s_* over the annual cycle. The annual total of soil respiration estimated from the logistic model was 645±5 g C m^−2^ year^−1^. The performance of the logistic model was poorest during periods of high soil temperature or low soil volumetric water content (VWC), which limits the model's ability to predict the seasonal dynamics of *R_s_*. The logistic model will potentially overestimate *R_s_* at high *T_s_* and low VWC. Seasonally, *R_s_* increased significantly and linearly with increasing VWC in May and July, in which VWC was low. In the months from August to November, inclusive, in which VWC was not limiting, *R_s_* showed a positively exponential relationship with *T_s_*. The seasonal sensitivity of soil respiration to *T_s_* (*Q_10_*) ranged from 0.76 in May to 4.38 in October. It was suggested that soil temperature was the main determinant of soil respiration when soil water was not limiting.

## Introduction

Forests store ∼45% of terrestrial carbon and are important components of the global carbon cycle. They absorb ∼30% of anthropogenic carbon emission from fossil fuel combustion and land-use change every year [1]. Carbon sequestration in forest ecosystems is determined by the difference between photosynthetic carbon fixation and ecosystem respiration. Soil CO_2_ efflux has been estimated to account for 60–90% of the total ecosystem respiration in temperate forests [2], [3], [4], [5], [6]. Understanding the carbon dynamics of soil respiration in different forest ecosystems and their responses to climatic factors is critical for estimating the future global carbon budget.

Soil respiration is often related to soil temperature [7], or soil temperature and soil water content [8], [9]. The temperature effect is due to its influences on microbial decomposition and root respiration. Low soil water content limits respiration by limiting microbial contact with available substrate and by causing dormancy and/or death of microorganisms [10]. On the other hand, high soil moisture limits gas exchange between soil and the atmosphere, thus leading to low soil oxygen concentration and restricting the aerobic respiration of the soil biocommunity. Soil respiration differs among ecosystems and varies with environmental conditions. Many studies have been done to quantify the soil respiration of different ecosystems and to understand the responses of respiration to environmental variables [6], [11], [12], [13], [14], [15].

Increasing forested land area through reforestation is one of the major strategies for mitigating carbon emissions. Globally, the area of new plantings of forests and trees is increasing by 2.8 million ha/year [16]. Within this global context, China's reforestation effort is significant because of the large area of new planting. It has been reported that the area of plantations increased by 8.43 million ha in the period 2004 to 2008 [17]. The resulting carbon uptake by plantations has been significant [18]. However, soils are the largest source of uncertainty in the terrestrial carbon balance of China [18], due to limited measurement of soil respiration and lack of repeated soil inventories. However, studies on soil CO_2_ flux in China's plantation forests are scarce. The few studies that have been done were mostly based on discontinuous measurements or those made at certain time points [19], [20], [21]. Measurements at certain time points are limited for understanding seasonal response of soil respiration to environmental factors [22].

In this study, soil CO_2_ flux was automatically and continuously monitored in a typical oriental arborvitae (*Platycladus orientalis*) plantation in the eastern part of Beijing, China, using an LI-8100A automated soil CO_2_ flux measurement system. *Platycladus orientalis* is one of most important species for reforestation in the temperate area in China [17]. The objectives were (1) to quantify the soil respiration of a typical plantation and (2) to understand the seasonal response of soil respiration to environmental factors.

## Materials and Methods

### Site description

The research was conducted in a 50-year-old plantation forest of oriental arborvitae (*Platycladus orientalis*) at Jiufeng Mountain (40°04′N, 116°06′E, 145 m a.s.l.), Beijing, China. The research site is owned by Beijing Forestry University. The field studies did not involve endangered or protected species and no specific permits were required for the described field studies.

The stand density was 1176 trees ha^−1^, with a mean tree height of 10.7 m and a mean tree diameter at breast height of 20.9 cm. The soil is loess type. The climate is temperate, with a mean annual temperature of 9°C and an average of 150 frost-free days per year. The mean annual precipitation is 600 mm, of which 70% falls in the period July-September, inclusive.

### Measurement of soil CO_2_ flux

Soil CO_2_ efflux was recorded continuously from May 1 to December 31 in 2008 using an LI-8100 automated soil CO_2_ flux measurement system with the 8100-104 long-term chamber (LI-COR Environmental, Lincoln, Nebraska USA). The flux was measured every 12 minutes. Five other soil collars were randomly and permanently placed in a 20 by 20 plot to reflect the spatial heterogeneity of soil respiration. The five collars were measured for five 12-min periods once every five days and the measurements were incorporated into the dataset for long-term chamber. Soil temperature and VWC near the chamber at 10 cm depth below ground were measured simultaneously using an 8150-203 temperature sensor and an 8150-202 soil water sensor (LI-COR Environmental, Lincoln, Nebraska USA), respectively. Measurements were recorded every 12 minutes.

### Data treatment and analysis

All 12-minute soil flux values greater than 30 µmol CO_2_ m^−2^s^−1^ or less than −10 µmol CO_2_ m^−2^s^−1^ were excluded. Then the values with a deviation from the mean greater than 5 times standard deviation were excluded over monthly time period [23]. These two steps resulted in 0.4% of respiration data being screened out. Mean hourly values were calculated based on 12–minute measurements and these mean values were used for analysis. Mean daily values were the average of hourly means over 24 hours. The missing soil temperature for annual *R_s_* estimation was filled using the standard method of the Fluxnet-Canada Research Network [24] and regression of soil temperature against year-round air temperature at the same site and Xianshan weather station (1 km away).

Five commonly used empirical models (Table 1) were used to regress soil respiration against soil temperature [23], [25]. The annual temperature sensitivity of respiration (*Q_10_*), the relative change in respiration for a 10°C change in temperature, was estimated using the logistic model, while the seasonal *Q_10_* was derived using the *Q_10_* exponential equation.

**Table 1:** Regression functions of soil respiration (*R_s_*) against soil temperature at 10 cm depth.

Name	Equation	*r^2^*	*RMSE*	*MEF*	Predicted *R_s_*(g C m^−2^)
Logistic	y = b_1_/(1+exp(b_2_(b_3_-x)))	0.81	0.6176	0.9474	386
Quadratic	y = b_1_+b_2_x+b_3_x^2^	0.82	0.617	0.9478	385
Log-transformed linear	ln(y) = b_1_+b_2_x	0.72	0.7586	0.9348	379
Exponential	y = b_1_b_2_^(x-10) /10^	0.8	0.6392	0.9413	391
Lloyd & Taylor	y = b_1_exp(-b_2_/(x+273.16+b_3_))	0.79	0.6614	0.9302	405

*RMSE, MEF,* and *r^2^* refer to root mean square error, model efficiency, and determination coefficient of regression, respectively. Predicted *R_s_* is the total of modeled values for period with measurements in comparison with observed total *R_s_* of 385 g C m^−2^.

### Statistical analysis

Regression analysis was used to examine the relationships between variables. Regression significance was evaluated using the F-statistic at a significance level of 0.05. The standard deviation for annual total *R_s_* was estimated using the Monte Carlo bootstrapping approach, in which hourly soil temperature was randomly sampled for 2000 times and the standard deviation of annual total computed. To test if the model-data fit is significantly different from the 1∶1 line, a bootstrapping analysis was performed with 1000 repetitions, and then test intercept  =  0 and slope  = 1 by t-test. All statistical analyses were done using Matlab (Version 7.12.0.635, The MathWorks, Natick, MA, USA).

To evaluate model performance, we use model efficiency (*MEF*) [26], [27], root mean square error (*RMSE*), and the coefficient of determination (*r^2^*) as the evaluation criteria.
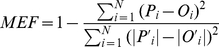
(1)where *P_i_* and *O_i_* are the corresponding predicted and observation values, *N* number of observations, *P_i_^'^* = *P_i_* – *ō*, *O_i_^'^* = *O_i_* – *ō* , *ō* the mean of all the observation values. *MEF* values range from [0,1] as agreement between predicted values and observations change from no agreement (*MEF*  = 0) to perfect agreement (*MEF*  = 1).

## Results

### Seasonal change in soil respiration

**Figure 1:**
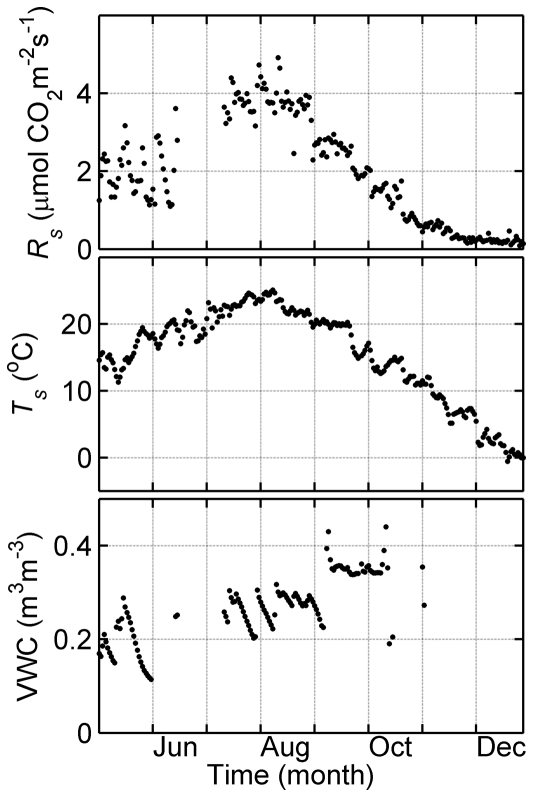
Daily mean of soil respiration (*R_s_*), soil temperature at 10 cm depth below ground (*T_s_*), and soil water content at 10 cm depth (VWC) from May to December in 2008.

Fig. 1 shows the mean daily soil respiration (*R_s_*), soil temperature (*T_s_*) and soil water content (VWC) from May 1 to December 31. *T_s_* ranged from −0.6°C on December 22 to 25.1°C on August 9. VWC ranged from 0.11 to 0.44 m^3^m^−3^, with the highest values in September. The period May to July was relatively dry, as seen in the low VWC (Fig. 1). *R_s_* ranged from 0.09 µmol CO_2_ m^−2^s^−1^ on December 9 to 4.87 µmol CO_2_ m^−2^s^−1^ on August 12. The seasonal pattern of *R_s_* was similar to that of *T_s_*, with *R_s_* increasing as the soil warmed in spring and summer, peaking in August and then declining in autumn to the lowest value in winter.

### Relationship between soil respiration and soil temperature

**Figure 2:**
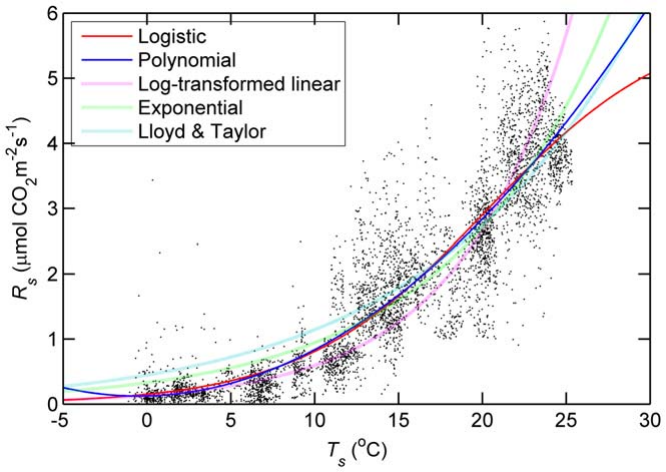
Hourly soil respiration (*R_s_*) as a function of soil temperature (*T_s_*) at 10 cm depth below ground. Data points were from May to December in 2008. Solid curves are fitted curves by equation listed in table 1.

Hourly soil respiration (*R_s_*) was significantly related to soil temperature (*T_s_*) for the May to December study period (Fig. 2), increasing with rising soil temperature. The *R_s_*-*T_s_* relationship was fitted using five commonly used empirical models including quadratic, logistic, log-transformed linear, exponential, and Lloyd & Taylor models (Table 1). The regression results are listed in Table 1. The logistic and quadratic models showed the best fit between *R_s_* and *T_s_*, having the highest *r^2^* and *MEF*, and the lowest *RMSE*.

**Figure 3:**
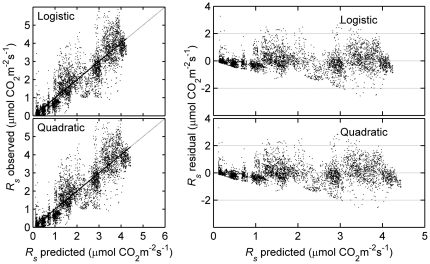
Comparison of measured hourly soil respiration (*R_s_*) as a fuction of modeled values using logistic and quadratic model for 2008.

Simulated hourly respiration rates from the logistic (*R_s_*__measured_ = −0.02 + 1.01* *R_s_* __modelled_, P<0.0001, *r^2^* = 0.82) and quadratic (*R_s_*__measured_ = 0.00 + 1.00 *R_s_* __modelled_, P<0.0001, *r^2^* = 0.82) models fit the measured values well (Fig. 3), but only the quadratic model gave a slope that was not significantly different from the 1∶1 line (intercept  = 0, P>0.05, slope  = 1, P>0.05). Residuals of soil respiration for both logistic and quadratic model exhibited a scatter of points that were uncorrelated with the fitted values (right panel in Fig. 3, P>0.05). The estimated annual *R_s_* from logistic and quadratic equations on the basis of gap-filled hourly soil temperature, was 645±5 g C m^−2^ year^−1^ and 642±5 g C m^−2^ year^−1^, respectively. Overall, both models similarly reflected the *R_s_*-*T_s_* relationship over most of observation time period. Annual temperature sensitivity of respiration (*Q_10_*) derived from logistic model was 4.1 over temperature ranging from −1.1 to 28.0°C.

### Seasonal response of soil respiration to soil temperature and water content

**Figure 4:**
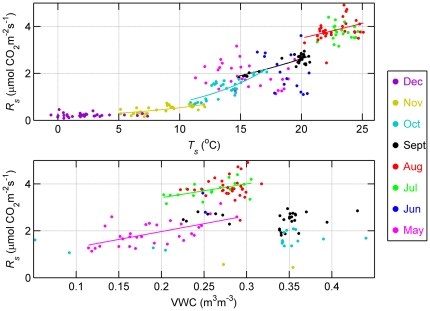
Seasonal relationships between soil respiration (*R_s_*) and soil temperature (*T_s_*) at 10 cm depth and soil water content (VWC) for each month in 2008. Data points were mean daily values.

Fig. 4 shows the seasonal changes in the response of *R_s_* to *T_s_* and VWC for each month from May to December. The curves shown are fitted curves with a statistically significant relationship between variables (P<0.05). Over a monthly time period, *R_s_* showed a significantly exponential relationship with *T_s_* in the months from August to November inclusive (P<0.05, *r^2^* = 0.14, 0.72, 0.65, and 0.49, respectively). In the months during summer from May 1 to July 31, *R_s_* was significantly and linearly related to VWC (P<0.05, *r^2^* = 0.39 and 0.33 in May and July, respectively), increasing with rising VWC. The responses of *R_s_* to environmental factors in June were not analyzed due to a large number of missing measurements of *R_s_* .

**Figure 5:**
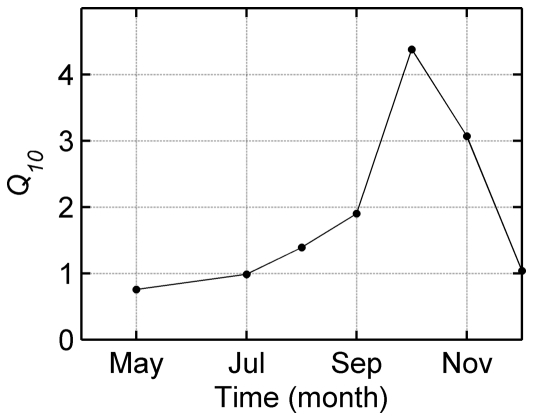
Seasonal sensitivity of soil respiration to soil temperature (*Q_10_*). Data points were derived from exponential regression over monthly time period as shown in figure 4.

The seasonal sensitivity of respiration to temperature (*Q_10_*) (Fig. 5), derived from the exponential model fitted over a monthly time period (Fig. 4), increased as the season proceeded, reaching the highest value in October and then declining gradually. *Q_10_* ranged from 0.76 in May to 4.38 in October.

## Discussion

Despite seasonal differences in the control of *T_s_* and VWC over *R_s_*, both temperature-only quadratic and logistic models accounted for 82% of the variation in hourly *R_s_*. Although the quadratic equation well fitted the data in measurement time period and expressed the dynamics of soil respiration at high temperature better than logistic model (Fig. 3), it did not fit the low-temperature data well and would potentially overestimate soil respiration at low temperature (Fig. 2). The logistic curve better reflects the dynamics of soil respiration at low temperature, both physically and physiologically. Therefore, the logistic model was judged to be superior. It seems that logistic model does not perform well in low soil moisture and higher temperature (May and June). Logistic model underestimates soil respiration by ∼25% in early summer (May and June), well estimates in summer time from July to October, and overestimates by ∼ 29% in fall or winter (November and December; Table 2). For periods with measurements, the predicted total (386 g C m^2^) of soil respiration was similar to observed total (385 g C m^2^) due to the overestimate under lower temperature offsetting underestimate in high temperature. The estimated annual *R_s_* of 645±5 g C m^−2^ in this study was comparable to those found in many temperate forest stands: 530 and 850 g C m^−2^ for a temperate mixed hardwood stand at two sites in Massachusetts [28], 710 g C m^−2^ for Norway spruce stand in Germany [29], 692–1472 g C m^−2^ for 11 mixed coniferous stands in China's Loess Plateau [21], and 438–598 g C m^−2^ for Scots pine [6].

**Table 2:** Comparison of measured mean soil respiration and corresponding modeled mean derived from the days with no occurrence of missing hourly value over 24-hour time period, and corresponding soil temperature (*T_s_*) and soil moisture (VWC) in 2008.

	May	Jun	Jul	Aug	Sept	Oct	Nov	Dec
*T_s_* (°C)	13.83(1.43)	17.03(1.32)	23.25(0.88)	22.78(1.31)	19.34(1.59)	13.63(1.64)	8.48(2.10)	2.47(2.07)
VWC (m^3^m^−3^)	0.22(0.04)	0.25(0.00)	0.26(0.03)	0.28(0.02)	0.33(0.05)	0.31(0.10)	0.31(0.06)	
Measured *R*_s_ (g C m^−2^day^−1^)	2.35(0.48)	2.89(0.00)	3.87(0.35)	3.98(0.46)	2.62(0.29)	1.38(0.44)	0.49(0.17)	0.22(0.06)
Modeled *R*_s_ (g C m^−2^day^−1^)	1.49(0.28)	2.21(0.00)	3.88(0.23)	3.76(0.34)	2.84(0.42)	1.46(0.33)	0.69(0.23)	0.26(0.11)

The monthly mean is based on daily mean values for both soil temperature and soil moisture and on daily total for soil respiration. Modeled soil respiration is derived from empirical logistic model (equation 1).

The annual sensitivity of soil respiration to temperature (*Q_10_*) of 4.1 over the time period from May to December, inclusive, was comparable to that of 4.0 for Scots pine in Finland [6], 3.2 for *Pinus tabulaeformis* in China's eastern part of Loess Plateau [21], 3.4–5.6 in a temperate mixed hardwood forest [26], 4.1 for Korean Pine in Changbai Mountain [30], and 3.9 for a boreal aspen [31]. It is higher than the mean *Q_10_* values reported for total ecosystem respiration, e.g. 2.4 for ecosystem on the global scale [32], the *Q_10_* of 2.0, which is typically used in modeling ecosystem respiration [33], and ∼1.4 across 60 FLUXNET sites [34]. Soil respiration may be more sensitive than total ecosystem respiration to temperature [34].

The seasonal changes in soil respiration were controlled by soil temperature and soil water content (Fig. 4), with soil water content being dominant in the months in early summer (before July), explaining ∼35% of the observed variation in respiration, and temperature being dominant in the months from August to November, explaining up to 72% of the variation. These results indicated that from September to November, inclusive, respiration was dominantly controlled by temperature when soil moisture was not limited (Fig. 1). A non-significant relationship between soil respiration and soil water content (P = 0.24), and its significant relationship with soil temperature with a lower determination coefficient in August (*R^2^* = 0.14, P<0.05), reflected a transition from water control over *R_s_* to temperature control.

Low soil moisture can limit *R_s_* by limiting microbial contact with available substrate and by causing dormancy and/or death of microorganisms [35]. These effects, in turn, reduce decomposition of soil organic matter, consequently reducing soil respiration. Besides, the sensitivity of respiration to temperature was accordingly reduced (*Q_10_* less than 1.5) in parallel with limiting microbial activity under low soil water content, thus resulting in a non-significant relationship between soil respiration and soil temperature in the months from May to July, inclusive. Curriel Yuste et al. reported that *R_s_* in a Scots pine stand in the Belgian Campine region decreased up to 50% when soil water content in the top 50 cm layer dropped below 0.15 m^3^m^−3^ [36], with soil water content largely controlling *R_s_*. Palmroth et al. found that soil respiration in an oak–hickory stand depended on only soil temperature when soil water content was over 0.20 m^3^m^−3^ [37], and on both soil temperature and water content when the soil was drier. It was also found that soil water stress decoupled *R_s_* and soil temperature in an 18-year-old temperate Douglas-fir stand with soil water content below the threshold of 0.11 m^3^m^−3^ [13].

One reason for observed low *Q_10_* at low soil water content is that water stress increases diffusion resistance and thus reduces contact between substrate and the extracellular enzymes and microbes involved in decomposition. Another reason for lower *Q_10_* under water stress conditions is decreased substrate supply [38], which in this case was likely due to (a) the drying out of the coarse fraction (litter) in the active surface layer, and (b) reduced photosynthesis, which decreases translocation of recent photosynthates to the rhizosphere [39], [40]. In laboratory incubation studies on semiarid soils, Conant et al. found that *R_s_* was generally related to soil temperature [41], but soil water deficit limited the positive relationship between *R_s_* and soil temperature, resulting in a significantly reduced response so that *Q_10_* declined with decreasing water content.

**Figure 6:**
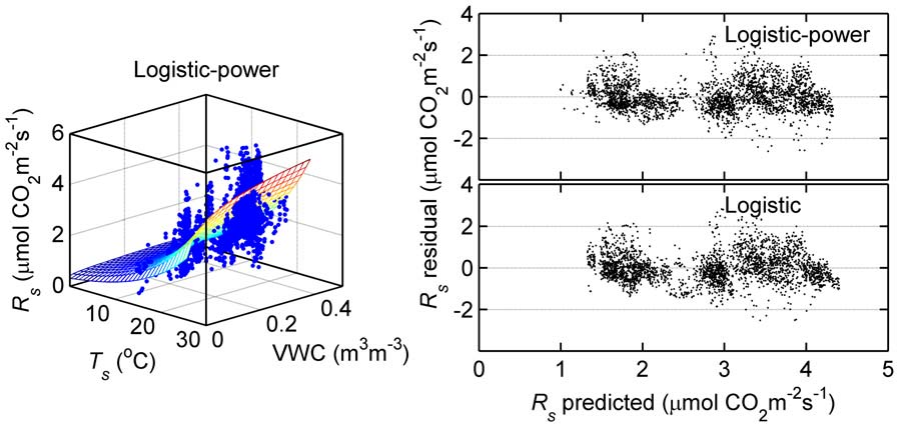
Soil respiration (*R_s_*) as a function of soil temperature (*T_s_*) and soil water content (VWC). Mesh is fitted using logistic-power function in table 3 and right panel is comparison of *R_s_* residuals from logistic-power and logistic functions.

Empirical models with two independent variables (*T_s_* and VWC) were fit to the data set with simultaneous measurement of *R_s_, T_s_* and VWC to reflect the effect of both factors [42]. A temperature logistic model that incorporated a power VWC relationship increased the *MEF* subtly and reduced *RMSE* slightly (Fig 6, Table 3). However, the analyses was limited by gaps in the VWC data. Further observations are needed to justify the use of a combined *T_s_* and VWC model.

**Table 3:** Comparison of combined soil respiration (*R_s_*) model (Logistic-power) with both soil temperature (*T_s_*) (°C) and soil water content (VWC) (m^3^m^−3^) as predictors and temperature-only model.

Name	Function	*r^2^*	*RMSE*	*MEF*	Predicted *R_s_* (g C m^−2^)
Logistic	*R_s_* = *b_1_*/(1+exp(*b_2_*(*b_3_*-*T_s_*)))	0.61	0.6934	0.8669	331.18
Logistic-power	*R_s_* = (*b_1_*/(1+exp(*b_2_*(*b_3_*-*T_s_*))))(VWC*^b4^*)	0.62	0.6827	0.8735	331.05

*RMSE, MEF,* and *r^2^* refer to root mean square error, model efficiency, and determination coefficient of regression, respectively. The values are from fitting functions for time period with measurements of *R_s_*, *T_s_* and VWC.

In conclusion, hourly soil respiration at the observed oriental arborvitae plantation exhibited a significant logistic relationship with soil temperature over the annual cycle. Seasonally, soil respiration showed a positively exponential relationship with soil temperature in the months from August to November and positively linear relationship with soil water content in months from May to July, a period during which soil water content was low and became a limiting factor. During the period of low VWC, the temperature-only models performed poorly. We conclude that an integrated model that incorporates both soil water content and soil temperature based on year-round measurements needs to be developed for a more accurate estimation of soil respiration in oriental arborvitae plantation and its contribution to the ecosystem carbon balance.
